# Childhood motor performance is increased by participation in organized sport: the CHAMPS Study-DK

**DOI:** 10.1038/s41598-019-54879-4

**Published:** 2019-12-12

**Authors:** Ann-Maree Vallence, Jeffrey Hebert, Eva Jespersen, Heidi Klakk, Christina Rexen, Niels Wedderkopp

**Affiliations:** 1 Discipline of Psychology, College of Science, Health, Engineering, and Education, Murdoch University, Perth, Australia; 2 Faculty of Kinesiology, University of New Brunswick, Fredericton, Canada; 3 Centre of Research in Childhood Health, Institute of Sports Science and Clinical Biomechanics, University of Southern Denmark, Odense, Denmark; 4 Department of Rehabilitation, Odense University Hospital, Institute of Clinical Research, University of Southern Denmark, Odense, Denmark; 5 University College Lillebaelt, Odense, Denmark; 6 University of Southern Denmark, Institute of Regional Health Research, Odense, Denmark; 7 Sports Medicine Clinic, Orthopaedic Department Hospital of Lillebaelt, Vejle, Denmark; 8 The Orthopedic Department, Hospital of South West Jutland, Esbjerg, Denmark

**Keywords:** Public health, Paediatric research

## Abstract

Evidence suggests that motor performance in children is declining globally. We tested whether participation in organized sport is associated with motor performance, and estimate the effect of 30 months participation in organized sport on motor performance. Study participants were 1067 primary school students, enrolled in the Danish Childhood Health, Activity, and Motor Performance School study. Participation in organized sport was reported via text messaging. Coordination-related motor performance composite, fitness-related motor performance composite, and total motor performance composite were calculated. Data were analyzed using Generalized Estimating Equations. Participation in organized sport was positively associated with motor performance (all composites) in models that did and did not control for baseline motor performance. For models that did not control for baseline motor performance, this equated to 2–6% increases in motor performance per weekly sport session; for models that did control for baseline motor performance, this equated to 1–5% increases in motor performance per weekly sport session. Positive associations between participation in organized sport and motor performance identify participation in organized sport as a way to improve motor performance in children. These results might provide the basis to determine whether participation in organized sport could be beneficial for children with developmental movement disorders.

## Introduction

Motor performance is a combination of fundamental motor skills and fitness-related components^1^. Motor performance is important to the development of physical, psychological, and social competencies in children and adolescents, and is likely a very important factor in developing an active lifestyle^1,2^. Motor performance is also likely an important factor in developing good sports-specific skills and physical activities^3,4^.

Links exists between physical activity levels and motor performance^4–6^. In children, motor performance is positively associated with physical activity, and inversely associated with sedentary activity^4^. Importantly, high levels of motor coordination at six years of age can buffer declining physical activity levels from age six to ten^7^. Identification of factors that increase motor performance in children is warranted given its importance in healthy development.

Childhood participation in organized sport is associated with higher levels of leisure-time physical activity^8,9^, greater time engaging in moderate-to-vigorous physical activity^10^, and an increased likelihood of meeting health-related physical activity recommendations^9^. Preliminary evidence supports a positive relationship between participation in organized sport and motor performance in children. First, using a cross-sectional design, participation in organized physical activity was positively associated with fundamental motor skill performance in 10–16 year olds^11^. Second, in a prospective longitudinal study, children with high motor competence had greater levels of participation in organized sport at two year follow-up than children with low motor competence^12^. Third, in a longitudinal study with two time points, children who reported participating in organized club sport had better motor coordination than children who partially participated or did not participate in organized club sport^13^.

Here, we aimed to investigate, with a prospective cohort design, the effects of organized sport on motor performance in primary school students. Specifically, we aimed to (i) determine whether participation in organized sport is associated with coordination- and fitness-related motor performance, and (ii) estimate the effect of 30 months of participation in organized sport on coordination- and fitness-related motor performance. It was expected that participation in organized sport would lead to up to 5% improvements in motor performance per weekly session of organized sport over the 30 month period.

## Methods

This is a prospective cohort design to investigate the effects of organized sport in primary school students using data from the Childhood Health, Activity, and Motor Performance School Study Denmark (CHAMPS-study DK). Nineteen primary schools in Svendborg, Denmark, were invited to participate; ten schools participated. Six schools had augmented physical activity programs, which offered 270 minutes of physical education lessons per week; four control schools offered 90 minutes of physical education lessons per week. Opportunities for physical activity during recess and in the classroom were not quantified.

Participation in organized sport before/after school and on weekends was measured over a 30 month period. Data were not collected for 6 weeks during summer holidays due to practical constraints^14^.

The CHAMPS Study-DK invited 1507 children aged 6–12 years to participate. Ethical approval for the CHAMPS Study-DK was provided by the Regional Scientific Ethical Committee of Southern Denmark (ID S-20080047) and methods were carried out in accordance with the human research ethics guidelines. Written informed consent was obtained from parents, and verbal informed consent was obtained from children prior to enrolment.

Participation in organized sport was reported by parents of participating children using mobile phone text messaging with a web-based system that automatically recorded text message responses (SMS-Track, Esbjerg, Denmark). This text message reporting system is reliable, comparable to phone interviews, and accepted by research participants^15^. Reporting was completed by parents because of concerns of the validity of children self-reporting^16^.

Each Sunday (weekly over the 30 month period), parents responded to the following question: *How many times did [CHILD] engage in organized sport during the last week?* Parents were required to respond with a number between ‘0’ and ‘8’: ‘0’ indicates that the child did not participate in organized sport in the previous week; ‘1’ to ‘7’ indicates the number of sessions of organized sport in the previous week; ‘8’ indicates that the child participated in >7 sessions of organized sport in the previous week. If participation in organized sport was reported, parents were asked to report the specific type(s) of sport and number of sessions. Sport type responses included soccer, handball, gymnastics, basketball, volleyball, swimming, horseback riding, and ‘other sports’. In the sport-specific analyses, we evaluated the effect of handball, soccer, and gymnastics on motor performance; we did not evaluate basketball, volleyball, swimming, or horseback riding because of low participation prevalence.

**Figure 1:**
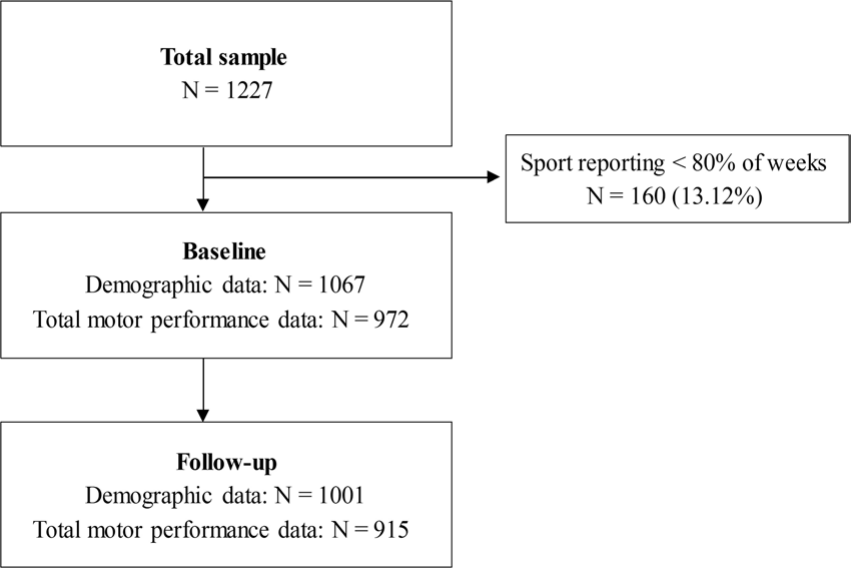
Study flow diagram.

If an inappropriate text message response was submitted, a researcher telephoned the parent for clarification. From the responses in the web-database, each child was categorized according to sport type and the number of sport sessions. We calculated the average total sport sessions per week over the 30 month period, and the average sport-specific sessions per week over the 30 month period. The study was “open”, allowing children moving to participating schools to enter the study, therefore, not all students participated from the start of the study. Participants with a response rate <80% were excluded from analyses. Figure 1 shows the study flow diagram.

Motor performance measures were obtained in school sports halls by research staff who underwent two days of training, including standardized instructions and measurement for each test, and standardized equipment calibration. In the training, clinicians and test personnel first practiced on each other and then on children aged 6–12 years (non-participants, the same age as CHAMPS Study-DK participants).

Six tests were used to assess motor performance: (i) Backward balance: a balance test from the Körperkoordinations Test für Kinder^17^ that requires participants to walk backwards on balance beams of three different widths (6, 4.5, and 3 cm). Participants complete three trials on each beam. The number of successful steps were counted (one point per step), with a maximum of 8 points per trial. Possible end scores range from 0–72 points. (ii) Precision throw: a precision test^18^ that requires participants to throw a ball at a target plate (with 3 zones, 0–3 points) three meters away. Participants complete two sets of five throws. Possible end scores range from 0–30 points. (iii) Shuttle run: an agility test from the Eurofit test battery^19^ that requires participants to complete five laps of a five meter course; run, pivot, return. Time taken to complete the five laps was recorded. (Note: a decrease in shuttle run time indicates an improvement in performance; we converted shuttle run scores to absolute values to assist with interpretation.) (iv) Vertical jump: corresponding to Abalakow’s vertical jump test^20^ requires participants to complete three vertical jumps; the highest jump was recorded (if the third jump was the highest, the participant continued to complete jumps until jump height reduced). Jumping height in cm was recorded. (v) Andersen test: a test of cardiovascular fitness^21^ that requires participants to intermittently run laps of a 20 meter course. Participants run for 15 seconds then rest for 15 seconds, for a total of 10 minutes. Distance covered in 10 minutes was recorded. (vi) Handgrip strength: a strength test from the Eurofit test battery^19^ that requires participants to complete two trials with a hand-held dynamometer using their dominant hand (JAMAR dynamometer, Scandidact, Cat No. 281128). Strength in kg was recorded (maximum score of the two trials).

Three composite motor performance scores were calculated: (i) total motor performance composite: average of all tests; (ii) coordination-related motor performance composite: average of backward balance, precision throw, shuttle run, and vertical jump; (iii) fitness-related motor performance composite: average of handgrip strength and Andersen test^22,23^. The dependent variables were the motor performance tests scores and the composite motor performance scores, and the independent variable was organized sport participation.

All analyses were performed using Stata v15.1 (StataCorp). We examined for associations between motor performance and organized sport participation using generalized estimating equation (GEE) models with an unstructured correlation matrix and robust standard errors. We constructed separate models for each of the composite and individual motor performance outcomes.

Sport participation was a continuous variable, estimated by the mean number of weekly sessions in *any* organized sport. In all models, sex, age, school type (i.e. schools that had augmented physical activity programs (offered 270 minutes of physical education lessons per week) and control schools (offered 90 minutes of physical education lessons per week)), and body mass index were included as covariates. When evaluating for sport-specific associations with the sports of interest—soccer, handball, and gymnastics—participation in other sports (i.e. not the sport of interest) were included as a covariates to adjust for participation in multiple sports. The reason for only assessing soccer, handball and gymnastics was that the other sports had too few participants to reliably assess the effect of participation on motor control. In addition, we could not assess multi-sport participation as we did not have information on the amount of participation in each of the sports, therefore, we could not reliably assess the correct effect of this participation on motor control.

We used GEE because this statistical technique is designed to take into account the inter-subjects variability by calculating the population average/marginal effects rather than the individual effects and the possible significant intraclass correlation in schools, classes, and subjects measured over time. The intraclass correlation was non-significant for schools (10^−12^ to 10^−16^), but significant for classes and subjects, thus we used as GEE. GEE treats the class and subject correlation structure as a ‘nuisance’ variable (as a covariate). We analyzed two multivariate models. The first model was used to investigate the association between sport and motor performance. Baseline motor performance *was not* included as a covariate in this model. The second model was used to investigate the effect of organized sport participation on change in motor performance over time; that is, to estimate the potential effect of participation in organized sport on the change in motor performance over 30 months. Baseline motor performance *was* included as a covariate in this model.

Variable normality and linearity of associations were examined graphically. To estimate the effect of 30-months of participation in organized sport on motor performance, continuous outcomes were reported with adjusted, unstandardized beta coefficients (β) and 95% confidence intervals representing the change in outcome per weekly sport session. We converted β to the percent difference relative to the baseline group mean to aid interpretation of the parameter estimates associated with participating in one sport session per week for the composite motor performance measures. Therefore, reported values represent a percentage change in motor performance per weekly sport session over the 30-month period. Alpha was set at 0.05 for all analyses.

## Results

The sample included data from 1,067 participants (53% female) with complete sport participation data (participants with a response rate <80% were excluded from analyses, as outlined above). Supplementary Table 1 (Supplementary Information) reports descriptive statistics for demographic and motor performance variables and Supplementary Table 2 (Supplementary Information) reports the prevalence and frequency of sport participation.

**Figure 2:**
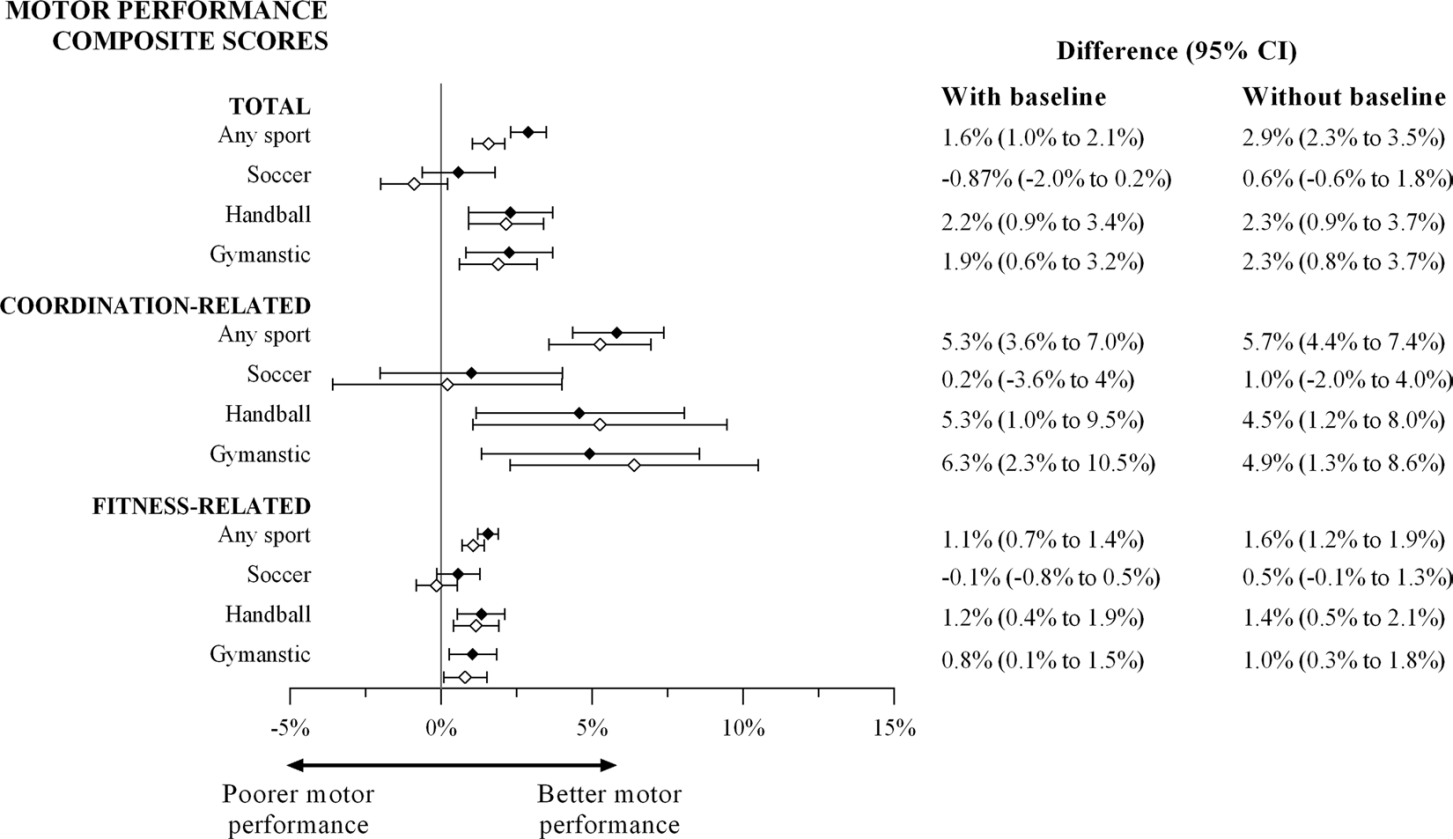
Average improvement in motor performance per weekly sport session with baseline motor performance included in the models as a covariate (open symbols) and without baseline motor performance included in the models as a covariate (filled symbols).

Participation in organized sport was associated with greater total motor performance, coordination-related, and fitness-related motor performance in models that did control for baseline motor performance (β range 0.16–0.31) and those that did not control for baseline motor performance (β range 0.23–0.56). For models that did control for baseline motor performance, which assessed the association between participation in organized sport and motor performance, this equates to 1–5% increases in motor performance per weekly sport session over the 30 month period. For models that did not control for baseline motor performance, which assessed the effect of participation in organized sport on change in motor performance over time, this equates to 2–6% increases in motor performance per weekly sport session over the 30 month period (Fig. 2).

When motor performance measures were examined separately, controlling for baseline motor performance, positive associations were found between organized sport participation and Anderson Test (β = 14.34), precision throw (β = 0.38), grip strength (β = 0.32), shuttle run (β = 0.29), and vertical jump (β = 0.41). No association was found between organized sport participation and balance (β = 0.10). When motor performance measures were examined separately, without controlling for baseline motor performance, positive associations were found between organized sport participation and balance (β = 0.62), Anderson Test (β = 18.76), precision throw (β = 0.50), grip strength (β = 0.40), shuttle run (β = 0.40), and vertical jump (β = 0.64). Figures showing the absolute changes in motor performance measures with participation in organized sport are presented in Supplementary Information (Figs. S1–S6).

### Soccer

Participation in organized soccer was not significantly associated with composite motor performance (analyses controlling for baseline motor performance: β range −0.17–0.01; analyses without controlling for baseline motor performance β range 0.06–0.11). Among individual motor performance measures, controlling for baseline motor performance, participation in organized soccer was not associated with balance, the Andersen Test, precision throw, grip strength, or shuttle run (β range −0.12–8.18) and was negatively associated with vertical jump (β = −0.91). In models that did not control for baseline motor performance, participation in organized soccer was positively associated with the Anderson Test (β = 14.45) and the shuttle run (β = 0.31) but was not associated with balance, precision throw, or grip strength (β range −0.13–0.72) and was negatively associated with vertical jump (β = −0.80).

### Handball

Participation in handball was positively associated with total motor performance, coordination-related, and fitness-motor performance (analyses controlling for baseline motor performance: β range 0.17–0.42; analyses without controlling for baseline motor performance β range 0.02–0.45). This equates to 1–5% increases in motor performance per weekly sport session over the 30 month period both for models that did and did not control for baseline motor performance (Fig. 2). Among individual motor performance measures, controlling for baseline motor performance, participation in organized handball was positively associated with the Andersen Test (β = 14.34) and precision throw (β = 1.30), but was not associated with balance (β = 0.66), grip strength (β = −0.03), shuttle run (β = 0.18), or vertical jump (β = −0.19). In models that did not control for baseline motor performance, participation in organized handball was positively associated with the Anderson Test (β = 15.30), precision throw (β = 1.39), and the shuttle run (β = 0.26) but was not associated with balance (β = 0.53), grip strength (β = 0.20) or vertical jump (β = −0.30).

### Gymnastics

Participation in gymnastics was positively associated with total motor performance, coordination-related, and fitness-motor performance (analyses controlling for baseline motor performance: β range 0.12–0.37; analyses without controlling for baseline motor performance β range 0.15–0.44). This equates to 1–6% and 1–5% increases in motor performance per weekly sport session over the 30 month period for models that did and did not control for baseline motor performance, respectively (Fig. 2). In models with baseline motor performance included as a covariate, participation in organized gymnastics was positively associated with balance (β = 2.09), the Andersen Test (β = 13.23), shuttle run (β = 0.25), but was not associated with precisions throw (β = 0.21), grip strength (β = −0.10) or vertical jump (β = 0.52). In models without baseline motor performance included as a covariate, participation in organized gymnastics was positively associated with balance (β = 2.23), the Andersen Test (β = 16.52), shuttle run (β = 0.33), but was not associated with precisions throw (β = 0.12), grip strength (β = −0.15) or vertical jump (β = 0.40).

The children excluded due to not reporting at least 80% of the weeks, were the same age, the same height, but heavier the mean weight being 34.3 kg at baseline, compared to 32.7 kg for the children complying with 80% rule for inclusion (p = 0.039), and the same was found for BMI, non-compliers 17.8 and complier 17.1. (p = 0.006).

## Discussion

We identified prospective positive associations between organized sport participation and improved motor performance in children. These positive associations were evident in analyses that did and did not control for baseline motor performance, with the adjustment for baseline resulting in little attenuation of the effect of participation in organized sport on motor performance. Together, these findings suggest that participation in organized sport might be an effective way to improve motor performance in primary school-aged children.

The current results show improved performance on both fundamental and fitness-related components of motor performance, including tests of balance, precision throw, vertical jump, shuttle run, grip strength, and Andersen test in children who participate in organized sport compared to those not participating in organized sport; results show that the positive associations equate to 2–5% increases in motor performance per weekly sport session over the 30 month period. (As an example of what this percentage change reflects, the effect of participation in organized sport on the Andersen test performance would be running 70 meter longer over the duration of the test (8%) in the group of children who participated in organized sport five times a week compared to children who did not participate in organized sport: see Supplementary Figs. S1–S6). Our results are consistent with preliminary evidence supporting a positive association between participation in organized sport and fundamental motor performance in children and adolescents^11,13^. The current results extend this previous research to show a positive association between participation in organized sport and fitness-related motor performance in children. Therefore, our results suggest that participation in organized sport in children is positively associated with motor performance across the coordination—fitness spectrum, not simply sport-specific motor performance.

In the current study, we examined change in motor performance over time controlling for baseline motor performance and therefore, taken together with the previous literature, the current results support the presence of an association between sports participation and motor performanceIn addition, by including baseline motor performance in analyses we examined the effect of sport participation over the 30 months that participation in organised sport was measured. It is important to note that controlling for baseline motor performance could bias the results: the children with highest level of motor performance are more likely to participate in sport, thus potentially making it more difficult to show an effect of organised sport participation on motor performance. However, this most likely means that the finding of increased motor performance with participation in organised sport in the current study is robust. This also fits with the suggestion that sports participation in early childhood promotes motor skill development, and that higher levels of motor skill competence provides a motor repertoire that enables participation in sports in older children, with these relationships strengthening over the lifespan^24^. It is important to note, however, that participation in organised sport in children decreases after 12 years of age^25^, so further investigation is required to better understand the relationship between participation in organized sport in children and physical activity levels in adulthood.

### Sport-specific effects of participation in organized sport

The complex and multi-faceted nature of the sport in which children participated seems to affect motor performance. For example, European handball requires coordination-related bimanual movements (i.e. precision throwing, catching) and bipedal movements (i.e. side-stepping). In the current study, children participating in organized handball had better motor performance on the Anderson Test and precision throw than children not participating in organized sport. (The effect of participation in organized handball fives time per week would be an increase in their score of 6 points for the precision throw (60%) compared to children who did not participate in organized sport; this increase in motor performance on the precision task could mean that the children participating in organized handball five times per week score 2 “bulls eye” compared to children who did not participate in organized sport not hitting this area on the target board at all: see Supplementary Figs. S1–S6.) Children who participation in organized gymnastics, which requires balance and agility, performed better in balance, the Anderson Test, and the shuttle run than children not participating in organized sport. Similarly, children participating in soccer performed better than children not participating in organized sport on the running-based motor performance measures, namely the Andersen Test and shuttle run; this is in line with the specific motor skills required for soccer, such as running speed and agility. It is important to note, however, that soccer was not positively associated with the total motor performance composite measure, or the either the fitness-related or coordination-related composite score. The reason that participation in organized soccer was only positively associated with the Anderson test and the shuttle run might be because only the lower limbs are engaged in soccer in a coordinated manner. Alternatively, it is possible that the children starting soccer already had high motor performance before joining, potentially due to hours spent in deliberate play in soccer, and therefore they could not improve motor performance further as soccer ‘only’ trains lower limb performance. It must be note, however, that these explanations are purely speculative. Taken together, these findings are consent with evidence that supports the development of sport-specific characteristics in motor performance, physical fitness, and anthropometry in children who participate in organized sport^26,27^.

### Effect of 30 months participation of organized sport on motor performance

We performed analyses controlling for baseline motor performance to estimate the effect of 30 months participation of organized sport on motor performance in children. These analyses showed that participation in organized sport was associated with 5 and 1% increases per sport session/week in coordination-related and fitness-related motor performance, respectively. Indeed, children participating in organized sport performed better than children not participating in organized sport (i.e. control group) for the Andersen Test, precision throw, grip strength, shuttle run, and vertical jump (ranging from 1–3% per sport session/week). Further analyses exploring the relationships between participation in specific organized sports and motor performance showed that both handball and gymnastics, but not soccer, was positively associated with motor performance when controlling for baseline motor performance (shown by the positive associations with total, fitness-related, and coordination-related composite scores). This suggests that participation in handball and gymnastics could lead to improvements in motor performance in primary school-aged children. The difference effect on motor performance between soccer and handball and gymnastics could be explained by the children starting sport with high motor performance competency, and only training lower-limb performance in soccer compared to both handball and gymnastics.

Given the association between motor performance and physical activity levels^5,6^ and healthy weight status in children^6,28^, improving motor performance could be beneficial for health. Recent evidence shows that motor performance in children predicts physical activity levels three years later^22^, and that early life motor performance predicts engagement with sport and exercise throughout life to older adulthood^29^. Our results complement this finding, showing a positive association between participation in organized sport and motor performance, which remains when controlling for baseline motor performance. A ‘motor proficiency threshold’ has been proposed, whereby children who reach or exceed the threshold are more likely to be physically active^4^. This hypothesis is based on the idea that a certain level of motor proficiency is necessary for children to learn the motor skills required for successful participation in sport^4,30^. While there is some evidence that, together with our study, supports the use of sport training to improve motor performance in healthy children^31^ and other studies in children with motor control impairments^32^, we would like to the results replicated.

It is important to note the limitation of the lack of process-oriented outcomes in the current study; future research should implement such outcomes, for example the *Test of Gross Motor Development*^33^. Additionally, a limitation of the current study is that we found BMI to be higher in the children not reporting more than 80% of the weeks; although we controlled for body mass index, this is an important consideration for future research as children with high body mass index might be might be less likely to participate in organized sport, and this might be the reason for the missing reporting. It is worth considering, however, that excluding the children with high BMI, who might not normally participating in organized sport, could make the current results even stronger: that is to say, the children with a high BMI likely had the lowest motor performance and excluding these children might make it more difficult to find a significant difference between those children participating in organized sport and those not participating in organized sport. Despite this, it is important to note that BMI does not reflect physiological fitness; heavier children will have poorer scores for speed and endurance tests than lighter children due to their weight but this does not mean that heavier children are physiologically less fit than lighter children. Future research should include measures of physiological fitness.

Although we accounted for several sources of potential confounders in our modelling, we were unable to control for all covariates with potential to influence the relationship between sport participation of motor performance. Furthermore, although we included sex as a covariate in the analysis there were more girls than boys in the current sample (53% girls). This is an important consideration given that some evidence shows girls drop out of organised sport at a younger age than boys^25,34^. Moreover, we quantified sport participation according to session frequency, but were unable to measure participation time within each session. In addition, in the current study, sport participation was a continuous variable, and when evaluating for sport-specific associations, participation in other sports (i.e. not the sport of interest) were included as a covariates. However, it is worth noting that children under 12 years of age who participate in more than 3 hours of organized sport per week could have better motor performance than children who participant in less than 3 hours of organized sport per week. To achieve this, the International Olympic Committee advises to “Encourage children to participate in a variety of different unstructured (i.e. deliberate play) and structured age-appropriate, sport-related activities and settings, to develop a wide range of athletic and social skills and attributes that will encourage sustained sport participation and enjoyment^35^”. But given that competition starts earlier in gymnastics than other sports, and gymnastics in youth often use greater variation in training (involving the whole body) compared to soccer, the advantage from participating in more than 3 hours of organized gymnastics per week could contribute to a greater improvement in motor performance per weekly sport session of gymnastics compared to soccer in the current study. This, however, is speculative and requires further investigation. Finally, it is important to note that we did not account for participants’ development over the course of the 30 month period. It is important for future research to investigate improvements in motor performance with participation in organized sport considering neurological and physiological development that is important for improvements in motor coordination and overall motor performance.

## Conclusions

The current results show that participation in organized sport in school-aged children is positively associated with motor performance across the coordination—fitness spectrum. In particular, participation in organized handball and gymnastics was positively associated with coordination-related and fitness-related motor performance. This suggests that participation in organized handball and gymnastics, or indeed any sport requiring coordination, agility, and fitness, could be an effective method for improving motor performance in children. It is important, however, to conduct a randomized controlled trial to better estimate the effect of organized handball and gymnastics on improved motor performance. The current results provide support for the use of organized sport to improve motor performance in typically developing children, and provide the basis to determine whether participation in organized children could be beneficial for children with movement disorders.

## Supplementary information

Supplementary information
