# Supplementary material for: Childhood motor performance is increased by participation in organized sport: the CHAMPS Study-DK
Source: Sci Rep. 2019 Dec 12;9:18920. doi: 10.1038/s41598-019-54879-4 (PMC6908570; doi:10.1038/s41598-019-54879-4)
Supplement: Supplementary file 1 — Supplementary information [file 41598_2019_54879_MOESM1_ESM.pdf]

Childhood motor performance is increased by participation in organized sport: the CHAMPS Study-DK.

Ann-Maree Vallence, PhD* [1], Jeffrey Hebert, PhD [1 2], Eva Jespersen, PhD [3 4], Heidi Klakk, PhD [3 5], Christina Rexen, PhD [3], Niels Wedderkopp, PhD MD [1 6 7]

Supplementary Information

Supplementary Table 1. Baseline and follow-up demographic, sport participation, and motor performance data.

	Baseline		Follow-up	
	N	Mean (SD)	N	Mean (SD)
Age (y)	1067	8.4 (1.4)	1001	10.9 (1.4)
Sex (N, % female)	1067	561, 52.6%	-	-
**BMI categories (N, %)**				
Normal		945 (92.9%)		945 (94.4%)
Overweight	1017	62 (6.1%)	1001	51 (5.1%)
Obese		10 (1.0%)		5 (0.5%)
**Motor performance composites**				
Total motor performance	972	15.1 (4.3)	915	17.2 (4.3)
Coordination-related motor performance	1007	11.1 (3.0)	980	12.9 (3.0)
Fitness-related motor performance	973	4.9 (1.6)	915	5.0 (1.6)
**Motor performance measures**				
Balance	1016	47.4 (13.1)	1001	51.0 (12.2)
Anderson Test	976	946.2 (103.1)	918	979.0 (105.7)
Precision throw	1018	13.5 (5.0)	1000	15.8 (4.3)
Grip strength	1017	17.6 (4.7)	999	21.0 (5.9)
Shuttle run	1007	23.7 (2.6)	981	22.5 (2.3)
Vertical jump	1017	28.9 (6.0)	990	33.4 (6.6)

[a] = Values are Mean (SD) unless otherwise indicated.

Supplementary Table 2. Prevalence and frequency of organized leisure-time sport participation.

	N	Participation (n, %)	Mean (SD)[a]
Sport participation			
Any sport	1067	1046, 98.0%	108.8 (70.4)
Soccer		747, 70.0%	19.8 (34.0)
Handball		656, 61.5%	16.1 (37.7)
Gymnastics		550, 51.5%	10.7 (30.2)

[a] Total sport sessions over 71 weeks among participating children.

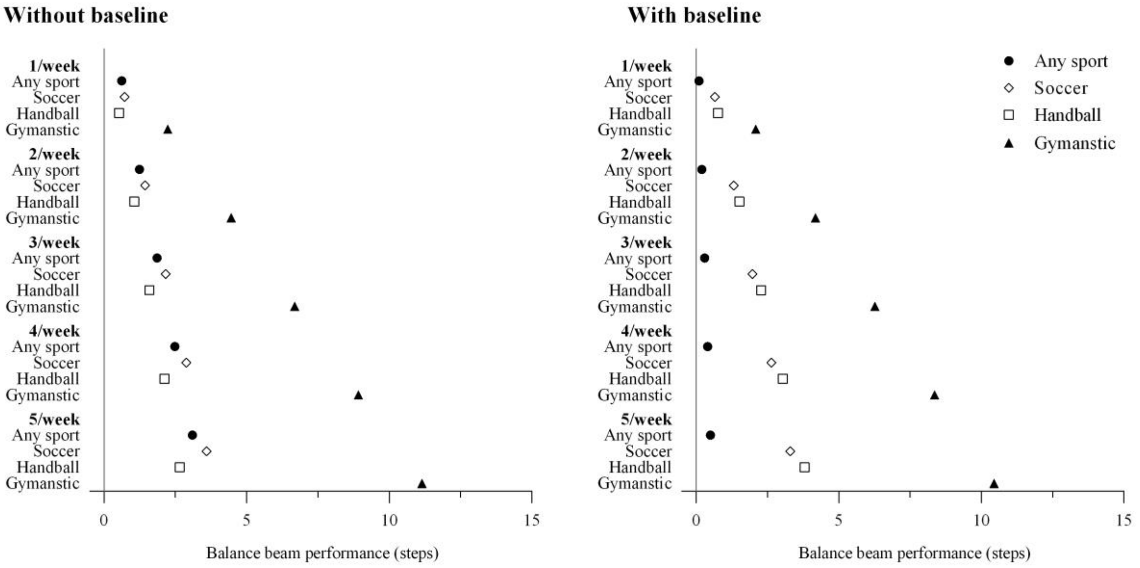

Supplementary Figure S1. Average change in balance beam performance (in steps) per weekly sport session without baseline motor performance included in the models as a covariate (left panel) and with baseline motor performance included in the models as a covariate (right panel).

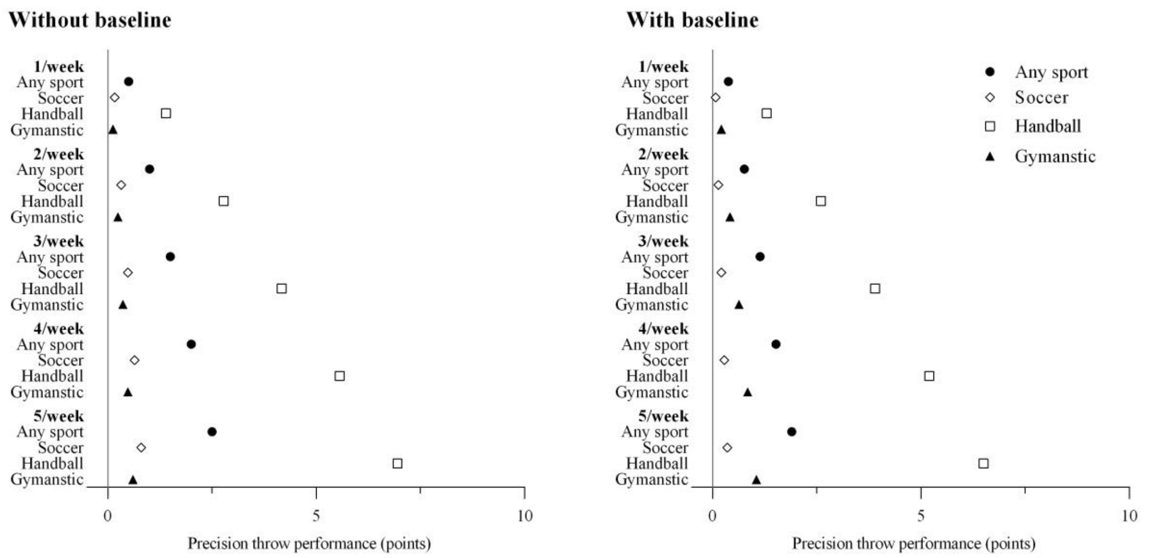

Supplementary Figure S2. Average change in precision throw performance (in points) per weekly sport session without baseline motor performance included in the models as a covariate (left panel) and with baseline motor performance included in the models as a covariate (right panel).

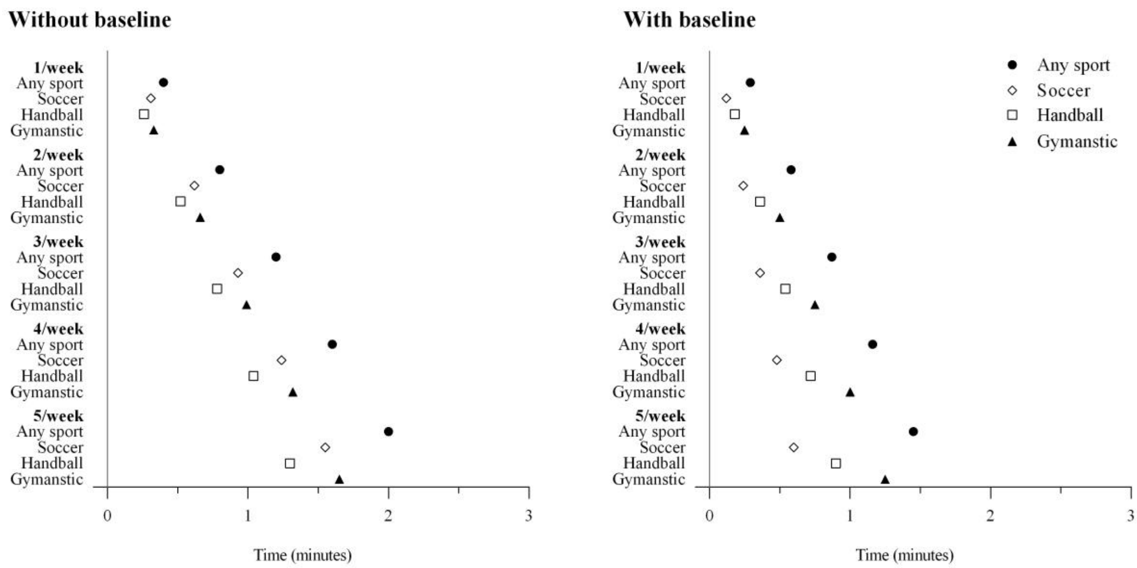

Supplementary Figure S3. Average change in shuttle run performance (in minutes) per weekly sport session without baseline motor performance included in the models as a covariate (left panel) and with baseline motor performance included in the models as a covariate (right panel).

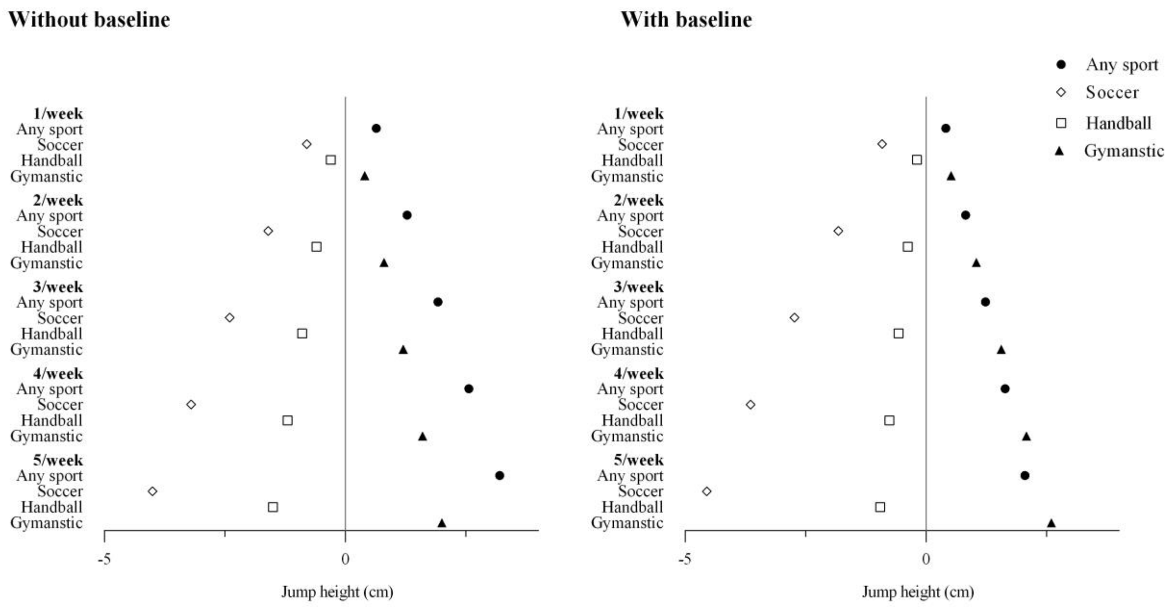

Supplementary Figure S4. Average change in vertical jump performance (in cm) per weekly sport session without baseline motor performance included in the models as a covariate (left panel) and with baseline motor performance included in the models as a covariate (right panel).

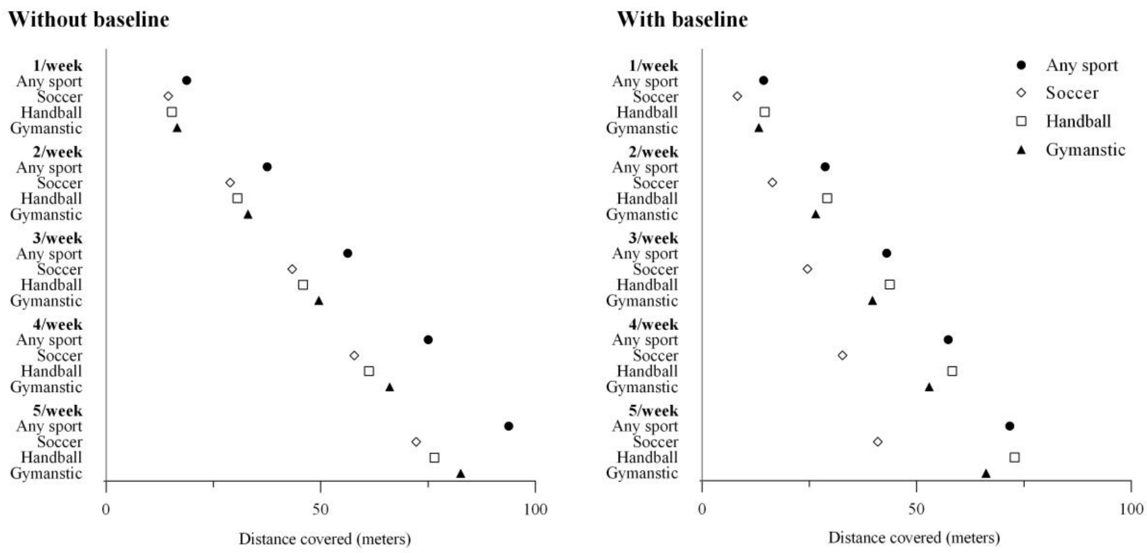

Supplementary Figure S5. Average change in Anderson test performance (in m) per weekly sport session without baseline motor performance included in the models as a covariate (left panel) and with baseline motor performance included in the models as a covariate (right panel).

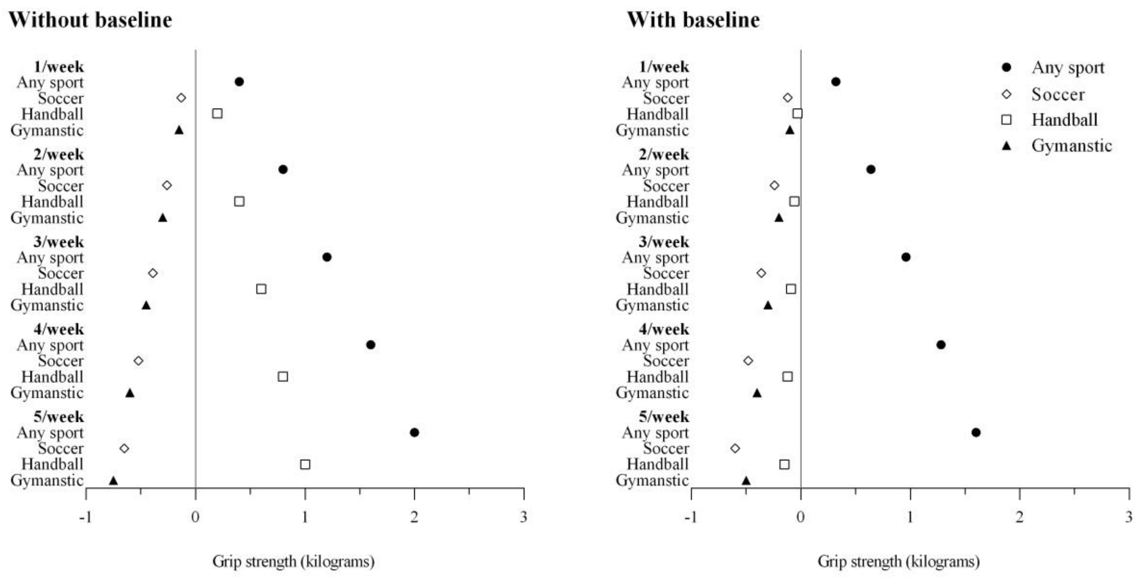

Supplementary Figure S6. Average change in grip strength (in m) per weekly sport session without baseline motor performance included in the models as a covariate (left panel) and with baseline motor performance included in the models as a covariate (right panel).
